# Presence of digestible starch impacts *in vitro* fermentation of resistant starch†

**DOI:** 10.1039/d3fo01763j

**Published:** 2023-12-06

**Authors:** Cynthia E. Klostermann, Martha F. Endika, Dimitrios Kouzounis, Piet L. Buwalda, Paul de Vos, Erwin G. Zoetendal, Johannes H. Bitter, Henk A. Schols

**Affiliations:** a Biobased Chemistry and Technology, Wageningen University & Research Wageningen The Netherlands; b Laboratory of Microbiology, Wageningen University & Research Wageningen The Netherlands; c Laboratory of Food Chemistry, Wageningen University & Research The Netherlands henk.schols@wur.nl; d Coöperatie Koninklijke AVEBE Veendam The Netherlands; e Immunoendocrinology, Division of Medical Biology, Department of Pathology and Medical Biology, University of Groningen and University Medical Centre Groningen The Netherlands

## Abstract

Starch is an important energy source for humans. Starch escaping digestion in the small intestine will transit to the colon to be fermented by gut microbes. Many gut microbes express α-amylases that can degrade soluble starch, but only a few are able to degrade intrinsic resistant starch (RS), which is insoluble and highly resistant to digestion (≥80% RS). We studied the *in vitro* fermentability of eight retrograded starches (RS-3 preparations) differing in rapidly digestible starch content (≥70%, 35–50%, ≤15%) by a pooled adult faecal inoculum and found that fermentability depends on the digestible starch fraction. Digestible starch was readily fermented yielding acetate and lactate, whereas resistant starch was fermented much slower generating acetate and butyrate. Primarily *Bifidobacterium* increased in relative abundance upon digestible starch fermentation, whereas resistant starch fermentation also increased relative abundance of *Ruminococcus* and *Lachnospiraceae*. The presence of small fractions of total digestible starch (±25%) within RS-3 preparations influenced the fermentation rate and microbiota composition, after which the resistant starch fraction was hardly fermented. By short-chain fatty acid quantification, we observed that six individual faecal inocula obtained from infants and adults were able to ferment digestible starch, whereas only one adult faecal inoculum was fermenting intrinsic RS-3. This suggests that, in contrast to digestible starch, intrinsic RS-3 is only fermentable when specific microbes are present. Our data illustrates that awareness is required for the presence of digestible starch during *in vitro* fermentation of resistant starch, since such digestible fraction might influence and overrule the evalution of the prebiotic potential of resistant starches.

## Abstract 2

Awareness is required for the presence of digestible starch when studying *in vitro* fermentation of resistant starch (RS). Such starch may steer and overrule the fermentation of RS, thereby affecting the evaluation of the prebiotic potential of RS.

## Graphical abstract

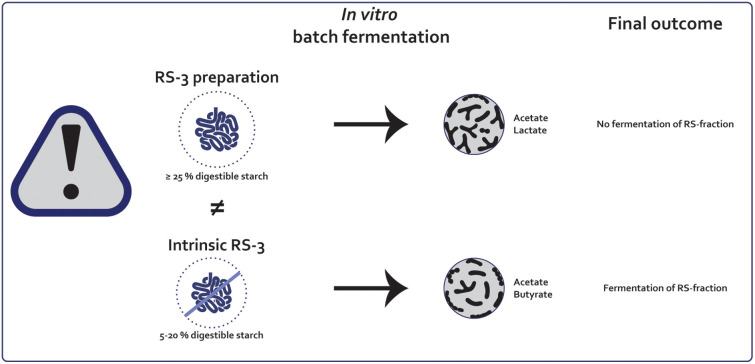

## 1. Introduction

Starch is an important energy source for humans. The major proportion of the starch we consume consists of digestible starch, that is partially digested by salivary α-amylase in the mouth and, depending on the pH, in the stomach and is further digested by pancreatic α-amylase in the small intestine to malto-oligomers.^1^ These malto-oligomers are hydrolysed by brush-border enzymes maltase-glucoamylase and sucrase-isomaltase to glucose^1^ and further metabolised to energy. If starch escapes digestion in the upper gastro-intestinal tract (GIT), it will transit to the colon where it will be fermented by gut microbiota to yield health-beneficial short-chain fatty acids (SCFAs).^2^ The amount of starch arriving in the colon depends largely on the digestion kinetics^3^ and is influenced by, among others, the type of starch, surrounding food matrices, transit time and age of the subject.^1,4–6^

A widely accepted model to study *in vitro* starch digestion in the upper GIT describes different fractions of digestible starch: rapidly digestible starch (RDS), which is digested within 20 min of a standardised incubation, and slowly digestible starch (SDS), which is digested between 20–120 min of incubation.^7^ The starch that is remaining after 120 min of digestion is considered resistant starch (RS).^7^ Nevertheless, this “RS” might be degradable using higher enzyme concentrations^8^ or longer incubation times^3^ and is therefore considered kinetically resistant starch. Such starches are different from intrinsic resistant starches, since these are not digestible by upper GIT enzymes.

Some starches are more resistant to upper GIT digestion than others due to their granular structure (type 2 RS) or conformation (type 3 RS (retrograded starches)) or due to a surrounding cell-wall matrix (type 1 RS) which is protecting the granular starches from pancreatic digestion.^4^ Also type 4 and type 5 RS are described, which resist digestion due to chemical modification or due to complexing with lipids, respectively.^4^ Although these five types of RS are defined and considered as “resistant starch”, most of them are partially digestible and only contain a fraction of RS.^9–12^ Previously, we made resistant starch type 3 (RS-3) preparations differing in molecular weight, molecular weight distribution and crystal type and found that the proportion of RDS, SDS and RS depended on these characteristics.^13^ Some of these RS-3 substrates were almost fully (>80%) resistant to digestion, did not change in physico-chemical properties due to digestion^13^ and even did not change in morphology due to digestion.^14^ Such RS-3 substrates are therefore considered intrinsic RS-3.^14^

To our knowledge, only few studies have been targeting the *in vivo* starch digestion in human directly.^9,15^ This is probably due to the use of invasive techniques that are needed to quantify released malto-oligomers and glucose in the small intestine. Starch digestion *in vivo* is mostly predicted indirectly by addressing the glycaemic response and comparing this to *in vitro* digestion assays.^16–18^ Since there is a lack of *in vivo* studies in human, it is still hard to predict how much of the digestible fraction of kinetically resistant starch will arrive in the colon. It has been speculated that a more rapid transit-time or starch-rich meals may result in the presence of digestible starch in the colon.^19^ A previous study supplementing subjects with 20 or 40 g day^−1^ digestible starch did not find changes in faecal microbiota composition after the intervention.^20^ This indicates that either all digestible starch had been absorbed in the small intestine or that the presence of digestible starch in the colon did not result in specific changes in microbiota composition. Animal studies that quantified remaining starch throughout the GIT showed that digestible starch had fully disappeared at the end of the small intestine, either by digestion or ileal fermentation.^21,22^

Although most of the digestible starch will be digested in the small intestine, still many studies discussing the fermentability of RS do not report the digestible fraction or remove this fraction prior to *in vitro* fermentation.^12,23–28^ Additionally, media used for *in vitro* fermentation, such as simulated ileal efflux medium (SIEM), routinely contain digestible starch.^29^ The presence of a large fraction of digestible starch in the colon of healthy adults is only likely when acarbose is used to block pancreatic digestion^30^ or originating from RS-1, in which otherwise digestible granules, such as present in wheat, rice or maize kernels, are protected by a cell-wall matrix and therefore not digested in the small intestine.^11^ Once the gut microbes open the cell-wall matrix, this starch is released and subject to fermentation. Digestible starch that is protected by a food matrix may also reach the colon. This depends on *e.g.* the microstructure,^31^ or the presence of dietary fibres that may slow down digestion.^32^ Although the impact of a digestible fraction on *in vitro* fermentation is not always clear, it should be realised that many different gut microbes are able to ferment soluble -digestible- starch due to the presence of starch-degrading glycoside hydrolase family 13 (GH13) enzymes in their genome^33^ (CAZy database^34^). In contrast, only a few microbes are reported to be able to degrade and ferment RS type 2 and 3 directly, such as *Ruminococcus bromii* and *Bifidobacterium adolescentis*.^35,36^ These microbes express, next to specific α-amylases, also specific carbohydrate binding modules (CBMs), such as CBM74, that are able to bind to insoluble starch particles^37^ and therefore accelerate hydrolysis.

The presence of a digestible fraction during *in vitro* fermentation is thus likely to affect the prediction of the prebiotic potential of RS, making pre-digestion to remove digestible starch necessary. Although *in vitro* digestion assays are developed to determine the amount of RS within a source and not necessarily as a pre-treatment prior to *in vitro* fermentation, many researchers used this pre-digestion treatment for studying the prebiotic potential of RS.^38–45^ Pre-digestion assays prior to *in vitro* fermentation have their weaknesses, such as inactivation of digestive enzymes without heat treatment, and drying of the obtained pre-digested fractions. Although some studies included digestible starch when studying *in vitro* fermentation of resistant starches,^12,23–28^ the impact of the presence of such a digestible starch fraction during *in vitro* fermentation by gut microbiota is not clarified in detail yet.

In the present study we determined the impact of digestible starch fractions as present within our RS-3 preparations on *in vitro* fermentation. These RS-3 preparations served as representatives of starches that contain a varying digestible fraction, although it needs to be confirmed if differences occur for other starches. Eight RS-3 preparations differing in rapidly digestible starch (RDS) content (grouped as ≥70% RDS, 35–50% RDS and ≤15% RDS) were inoculated with a pool of four healthy adult faecal samples and subsequently, the starch degradation, SCFA production and changes in microbiota composition were determined at different incubation times. To investigate a possible generic impact of gut microbiota on the fermentability of digestible *versus* resistant starch, we also incubated one fully digestible starch (from the group ≥70% RDS) and two intrinsic RS-3 substrates (from the group ≤15% RDS) with faecal inocula of two adults and two weaning infants at six and nine-ten months old and quantified the SCFAs produced.

## 2. Materials and methods

### 2.1. Materials

l-Cysteine hydrochloride, 2-(*N*-morpholino) ethanesulfonic acid (MES) and soluble potato starch (SPS) were obtained from Sigma-Aldrich (St Louis, Missouri, USA).

#### 2.1.1. RS-3 preparations

RS-3 preparations were prepared by crystallizing α-1,4 glucans obtained by either debranched (enzymatically modified) amylopectins or by enzymatic synthesis.^13^ The (enzymatically modified) amylopectins included waxy potato starch (Eliane100) and highly branched starch of potato (*M*_w_ ± 100 kDa, 8% branch points) provided by AVEBE (Veendam, The Netherlands) and waxy rice starch (Remyline XS) obtained from Beneo (Mannheim, Germany). The physico-chemical characteristics of the RS-3 preparations and the *in vitro* digestibility were described previously by Klostermann *et al.* (2021)^13^ and are summarised in Table 1 in the Results section.

**Table 1:** Characteristics of RS-3 preparations used in this study, as described in Klostermann *et al.* (2021)^13^

Name	Reported as^13^	DPn	PI	Crystal type	RDS (%)	SDS (%)	RS (%)	RDS group
P14-A	dHBPS-A	14.3	1.33	A	40.1 ± 5.4	44.7 ± 8.9	15.2 ± 3.6	35–50%
P14-B	dHBPS-B	14.0	1.35	B	92.1 ± 12.0	7.6 ± 1.8	0.2 ± 10.3	≥70%
N15-B	sG2-B	15.2	1.25	B	69.6 ± 4.2	19.9 ± 2.0	10.5 ± 2.2	≥70%
P22-B	dWRS-B	21.9	1.50	B	38.2 ± 2.2	41.9 ± 0.7	19.9 ± 2.4	35–50%
N18-A	sG5-A	18.0	1.21	A	12.4 ± 9.1	−0.3 ± 6.1	88.0 ± 3.8	≤15%
N18-B	sG5-B	18.0	1.21	B	50.7 ± 0.4	23.4 ± 3.0	25.9 ± 3.3	35–50%
P40-B	dWPS-B	39.9	2.11	B	15.3 ± 1.7	10.5 ± 1.9	74.2 ± 2.0	≤15%
N76-B	sG65-B	75.6	1.07	B	3.2 ± 0.9	2.6 ± 0.2	94.2 ± 0.8	≤15%

#### 2.1.2. Faecal slurry

Faecal material of four healthy adults was collected, after signing a written informed consent. Subjects were between 27 and 35 years old, had a BMI between 19–22 kg m^−2^, were non-smokers, did not have any health complaints, and did not use antibiotics for over 6 months prior to collection. After defecation, the faeces was stored immediately in a sterile 50 mL tube with a filter screw cap (Greiner Bio-One CELLSTAR™ tube, Kremsmünster, Austria). The tube was placed inside a pouch with a BD GasPak EZ anaerobe gas generating system with indicator (BD Diagnostics, Sparks, Maryland, USA) and stored at 4 °C for a maximum of 24 h until processing. Pooled adult faecal slurry was prepared in an anaerobic chamber (gas composition 4% H_2_, 15% CO_2_, 81% N_2_, Bactron 300, Sheldon Manufacturing, Cornelius, Oregon, USA), by mixing equal aliquots of faeces with a pre-reduced dialysate-glycerol solution to 25%w/v as previously described and validated.^46^ The pre-reduced dialysate-glycerol solution contained ten times diluted dialysate solution (25 g L^−1^ K_2_HPO_4_·3H_2_O, 45 g L^−1^ NaCl, 0.05 g L^−1^ FeSO_4_·7H_2_O and 0.5 g L^−1^ ox-bile) (Tritium Microbiologie, Eindhoven, The Netherlands), 10% glycerol, 0.5 mg mL^−1^ resazurin, 0.4 g L^−1^l-cysteine–HCl, 0.45 g L^−1^ CaCl_2_, and 0.5 g L^−1^ MgSO_4_·7H_2_O. The final faecal slurry was snap-frozen in liquid nitrogen and stored at −80 °C prior to use. Faecal samples of infants were collected within the Baby Carbs Study as briefly reported by Endika and co-authors.^47^ Faecal slurries of two individual adults, two weaning infants at 6 months old and the same two infants at 9–10 months old were prepared as described above, snap-frozen in liquid nitrogen and stored at −80 °C prior to use.

### 2.2. *In vitro* batch fermentation of RS-3 preparations

*In vitro* batch fermentations were performed at substrate concentrations of ±10 mg mL^−1^, which is a widely used substrate concentration in such studies.^24,48,49^ The amount of resistant starch that might arrive in the colon when consuming 250 grams of boiled potatoes (1 dose) might be around 1 mg mL^−1^. Hereby, we assume a total starch content of ±84% (dry weight) of which 3.4% resistant starch^50^ and an upper GIT volume of 2 L. Taking into account that additional dietary fibre would be present in the diet and maybe even some digestible starch might escape digestion, our substrate concentration (10 mg mL^−1^) fits within a dose of physiological relevance.

The culture medium was based on Simulated Ileal Efflux Medium (SIEM) as described by Minekus *et al.* (1999)^29^ with minor modifications as reported previously.^14^ Especially the carbohydrate component within modified SIEM (mSIEM) was lowered to 0.592 g L^−1^. Culture medium containing 11.11 mg mL^−1^ soluble potato starch (mSIEM + SPS) was prepared as described previously.^14^

The *in vitro* batch fermentations were performed as described previously.^14^ In short, ±20 mg (dry weight) RS-3 preparations were weighed in duplicate in sterile 5 mL serum bottles for each individual sampling time. The weighed RS-3 preparations were stored overnight in the anaerobic chamber.

In the anaerobic chamber, inoculum was prepared by diluting pooled adult faecal slurry to 10 mg mL^−1^ faeces in mSIEM. An aliquot of 1.8 mL mSIEM was added to the serum bottles containing RS-3 preparations and 0.2 mL inoculum was added to reach final substrate concentrations of ±10 mg mL^−1^. Similarly, an aliquot of 1.8 mL mSIEM + SPS was added to empty serum bottles and 0.2 mL inoculum was added. In addition, substrate blanks (without inoculum) and medium blanks (without additional substrate) were included. The serum bottles were capped with butyl rubber stoppers and incubated at 37 °C, 100 rpm for 0, 24 and 48 h.

At each time point, the serum bottles were decapped and the contents were transferred to Safe-Lock Eppendorf tubes (Eppendorf, Hamburg, Germany). The tubes were centrifuged, the pellet and supernatant were separated and further processed as described previously.^14^ The pH was measured using non-bleeding pH indicator strips 4.0–7.0 (Merck, Darmstadt, Germany). The supernatant was heated in a Safe-Lock Eppendorf tube to 100 °C, 800 rpm for 10 min in an Eppendorf shaker (Eppendorf) and stored at −20 °C until analysis. The pellet was snap-frozen with liquid nitrogen, stored at −80 °C and freeze-dried.

In a second, independent fermentation experiment, we investigated the fermentability of SPS and two intrinsic RS-3 substrates (N18-A and N76-B) using individual faecal inocula obtained from adults and infants (Information S1†).

### 2.3. Total starch quantification of fermented RS-3 preparations

Starch was quantified in the supernatant and in the freeze-dried pellets obtained after fermentation using the Megazyme Total Starch Kit (AA/AMG) (Megazyme, Wicklow, Ireland), according to the company protocol and adjusted for smaller sample sizes as described by Klostermann *et al.* (2023).^14^

### 2.4. Short-chain fatty acids and other organic acids produced by fermentation

SCFAs, lactic and succinic acid were analysed using HPLC-RI/UV as previously described.^14^

### 2.5. Microbiota composition & analysis

Microbiota composition was determined as described previously^14^ in all biological duplicates, except for SPS t0 since only 1 freeze-dried pellet contained enough DNA to perform 16s ribosomal RNA (rRNA) gene sequencing. In short, the V4 region of the 16S rRNA gene of purified DNA was amplified in duplicate using barcoded primers. The PCR products were purified, pooled and sent for Illumina Hiseq2500 (2 × 150 bp) sequencing (Novogene, Cambridge, UK). Raw sequence data of the 16S rRNA gene amplicons was processed using the NG-Tax 2.0 pipeline with default settings.^51^ Taxonomy of each amplicon sequence variant (ASV) was assigned based on the SILVA database version 138.1.^52,53^ Data was analysed using R version 4.1.0 and the R packages phyloseq version 1.38.0,^54^ microbiome version 1.17.42^55^ and microViz version 0.10.1^56^ as previously described.^14^ Principle component analysis (PCA) and principle coordinate analysis (PCoA) were used to visualise the microbiota variation between substrates after centered-log-ratio (CLR) transformation or Generalised UniFrac distances (*i.e.* taking phylogenetic relatedness and relative abundance of taxa into account, with an extra parameter α controlling the weight of abundant lineages^57^) and Unweighted UniFrac distances (*i.e.* taking phylogenetic relatedness between taxa, but no relative abundance of taxa into account), respectively. PERMANOVA analyses were performed to determine if the % RDS, % SDS and % RS (as a continuous variable) present within RS-3 preparations significantly influenced the microbiota composition. Sequences and sample information has been submitted to the European Nucleotide Archive of the European Bioinformatics Institute (EBI). The data can be found under the study accession number ‘PRJEB64324’. ENA Browser (ebi.ac.uk) (https://www.ebi.ac.uk/ena/browser/view/PRJEB64324).

### 2.6. Data analysis

The obtained data were subjected to analysis of variance (ANOVA) using R version 4.3.1 (R Core Team). For that, SPS and RS-3 preparations were grouped per RDS content (%): group 1, ≥70% RDS: SPS, P14-B, N15-B; group 2, 35–50% RDS: P14-A, N18-B, P22-B; group 3, ≤15% RDS: N32-B, P40-B, N76-B. The observations per RDS group (*n* = 6) were used to model the effect of RDS content and fermentation time (24 h, 48 h) as well as their two-way interaction on total starch recovery and total organic acid formation. Normality of data residuals and homogeneity of variance were additionally checked. To test the significance of the differences between RDS groups, Tukey's *post-hoc* test was performed, with a significance threshold set at *p* < 0.05.

## 3. Results

### 3.1. RS-3 preparations

We investigated the impact of the presence of a digestible starch fraction of nine different starches on *in vitro* fermentation characteristics, such as starch degradation, SCFA formation and microbiota composition. Eight starches studied were so-called RS-3 preparations while soluble potato starch (SPS) was taken as a positive control. The RS-3 preparations were prepared from α-1,4 glucans of different average *M*_w_, obtained either by debranching amylopectins of different sources or by enzymatic synthesis. The obtained α-1,4 glucans were crystallised in A- or B-type polymorphs. To determine the fractions of rapidly and slowly digestible starch (total digestible starch) and resistant starch, the samples were *in vitro* digested using porcine pancreatin and amyloglucosidase.^13^ The overall characteristics of the eight RS-3 preparations are shown in Table 1. The sample codes are named after the RS-3 physico-chemical characteristics for intuitive understanding with N: narrow disperse, P: polydisperse, number: DPn, A: A-type crystal, B: B-type crystal.

The RS-3 preparations obtained from debranched amylopectins had a more polydisperse *M*_w_ distribution (PI ≥ 1.3), whereas those obtained from enzymatically synthesised α-1,4 glucans had a more narrow disperse *M*_w_ distribution (PI ≤ 1.25). After *in vitro* digestion, the RS-3 preparations and SPS were grouped based on their RDS content. These groups consisted of samples having ≥70% RDS (SPS, P14-B, N15-B), 35–50% RDS (P14-A, N18-B, P22-B) and samples having ≤15% RDS (P40-B, N18-A and N76-B). Especially N18-A and N76-B contained a very low amount of total digestible starch (RDS + SDS) and were therefore considered intrinsic RS-3. Some RS-3 preparations with similar *M*_w_ and *M*_w_ distribution but differing in crystal type fell into different groups based on their digestion properties (P14-A *vs*. P14-B, N18-A *vs*. N18-B).^13^ Together, these eight RS-3 preparations and SPS represent a broad collection of starches differing in contents of RDS, SDS and RS, which allows us to study the effect of a digestible fraction on *in vitro* fermentation of RS-3 preparations.

### 3.2. Starch having a digestible fraction is fermented differently compared to intrinsic resistant starch

The RS-3 preparations and SPS were fermented *in vitro* during 48 h using an inoculum of a pool of four healthy adult faecal samples. The starch was quantified based on the presence of soluble starch in the supernatant and insoluble starch in the obtained pellet after incubation with (Table S1†) and without faecal inoculum (Table S2†). This revealed that some RS-3 preparations solubilised during 24 h of incubation (Table S2†) and that these soluble fractions were not detected in the presence of the faecal inoculum (Table S1†), indicating that the soluble fraction was fermented prior to the insoluble fraction. Especially P14-B solubilised for ±70% after 24 h of incubation, but also other RS-3 preparations like N15-B and P14-A solubilised for ±30% after 24 h of incubation (Table S2†), which is likely caused by the short α-1,4 glucan chain length of these RS-3 preparations. Previously, it has been shown that especially short α-1,4 glucans present within recrystallised starch tend to solubilise when heated; and when present in B-type crystallites these short α-1,4 glucans tend to solubilise faster than when present in A-type crystallites,^58^ clearly confirming our observations.

It should be noted that insoluble starch was hard to quantify accurately since sampling of insoluble starch had some limitations (*i.e.* the pellets obtained after centrifugation were very unstable), especially for samples at t0 and samples without inoculum. When fermentation proceeded, the biomass together with the insoluble starch particles made quantification easier and more reliable. To achieve a useful comparison among fermentation rates of RS-3 preparations by gut microbiota, we normalised the obtained starch recovery during fermentation for total starch content at t0.

To have a first indication of the influence of RDS content on the fermentation behaviour of RS-3 preparations, the latter were grouped on the basis of RDS content (Table 1: ≥70% RDS, 35–50% RDS, ≤15% RDS) and the total starch recovery and total organic acid formation after incubation were determined (Table 2). The significant two-way interaction for total starch recovery (*p* < 0.05) and the trend to significance for total organic acid release (*p* < 0.1) demonstrated that both the starch type (≥70% RDS, 35–50% RDS, ≤15% RDS) and the fermentation time (24 or 48 h) influenced the extent of starch fermentation and formation of metabolites. RS-3 preparations containing ≥70% RDS were almost fully degraded within 24 h (Table 2). RS-3 preparations containing 35–50% RDS were degraded for ±70% after 24 h of incubation, after which degradation proceeded until ±16% was left after 48 h of incubation. RS-3 preparations containing a minor amount of RDS (≤15%) were degraded for ±23% during 24 h, after which degradation proceeded until 44% of starch was left after 48 h of incubation. RS-3 preparations containing ≤15% RDS were thus degraded slower and to a lower extent, compared to RS-3 preparations containing ≥35% RDS.

**Table 2:** Starch degradation and total organic acids (acetic, propionic, butyric and lactic acid) production after *in vitro* batch fermentation of SPS and RS-3 preparations having rapidly digestible starch (RDS) content ≥70% RDS: SPS, P14-B, N15-B; 35–50% RDS: P14-A, N18-B, P22-B; ≤15% RDS: N32-B, P40-B, N76-B, with pooled adult faecal inoculum. Values within the same column not sharing common notation differ significantly (*p* < 0.05)

RDS group	Fermentation time (h)	Total starch recovery (%)	Total organic acid (μmol mg^−1^)
≥70%	24 h	5.1^d^	13.2^a^
	48 h	2.5^d^	13.7^a^
35–50%	24 h	30.4^bc^	10.0^b^
	48 h	16.2^cd^	12.5^ab^
≤15%	24 h	77.0^a^	3.6^d^
	48 h	43.9^b^	6.7^c^
SEM^a^		4.01	0.577
*p*-Values	RDS group	<0.001	<0.001
	Fermentation time	<0.001	<0.001
	RDS group × fermentation time	0.003	0.082

a Standard error of the mean, *n* = 6.

After 24 h of incubation, approximately 13.2 μmol mg^−1^ total organic acids were formed for RS-3 preparations containing ≥70% RDS (Table 2). The total organic acid content did not increase significantly between 24 and 48 h (*p* > 0.05), indicating that fermentation was already complete after 24 h of incubation for these substrates. For RS-3 preparations containing 35–50% RDS, the total amount of total organic acids increased between 24 and 48 h of incubation to 12.5 μmol mg^−1^. RS-3 preparations containing ≤15% RDS increased from 3.6 to 6.7 μmol mg^−1^ between 24 and 48 h of incubation and were fermented to significantly lower amounts of total organic acids compared to RS-3 preparations containing ≥35% RDS (*p* < 0.05). The increase in formation of total organic acids was in line with the decrease in starch recovery for all different RDS groups. These findings emphasize the importance of RDS content for the *in vitro* fermentation of RS-3 preparations.

**Fig. 1:**
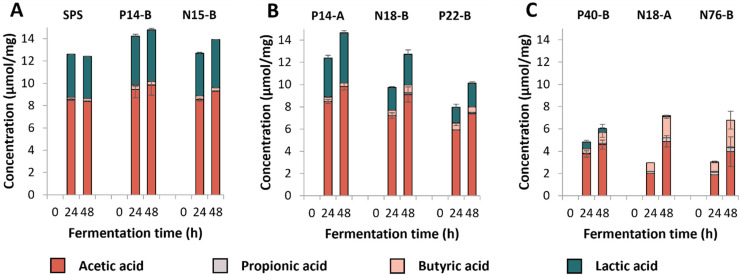
SCFA and lactic acid formation (μmol mg^−1^ substrate) during 48 h of fermentation of RS-3 preparations by pooled adult faecal inoculum. Figure A, B and C represent RS-3 preparations containing ≥70% RDS, 35–50% RDS or ≤15% RDS, respectively. The average of biological duplicates is shown.

**Fig. 2:**
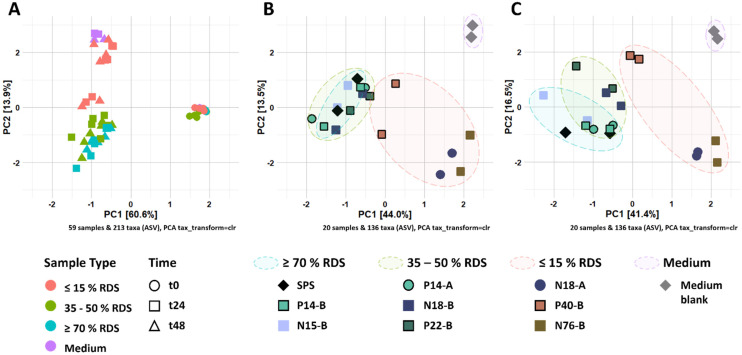
(A) Ordination plot of CLR-transformed relative abundances of ASVs from fermented RS-3 preparations by pooled adult faecal inoculum over time. Figure B and C refer to CLR-transformed relative abundances of ASVs at t24 and t48, respectively.

Next, we investigated in-depth starch degradation (Fig. S1†), individual SCFA formation (Fig. 1) and microbiota composition (Fig. 2) during *in vitro* fermentation of structurally distinct RS-3 preparations (Table 1).

The individual RS-3 preparations containing ≥70% RDS (SPS, N15-B and P14-B) all were completely fermented within 24 h of incubation (Fig. S1A†). Samples containing 35–50% RDS (P14-A, N18-B, P22-B) were degraded for 60–80% during the first 24 h of incubation (Fig. S1B†). The starch degradation in these samples continued up to 48 h of incubation, but degradation rate slowed down for N18-B and P22-B. P14-A was degraded to a larger extent than N18-B and P22-B, probably due to the presence of a larger total amount of digestible starch within P14-A, compared to N18-B and P22-B (Table 1). RS-3 preparations having ≤15% RDS (N18-A, N76-B, P40-B) behaved differently during 48 h of incubation (Fig. S1C†). Intrinsic RS-3 with a narrow disperse *M*_w_ distribution (N18-A and N76-B) were degraded at a similar rate with ±40% starch remaining after 48 h, whereas P40-B (polydisperse *M*_w_ distribution) showed a different degradation pattern with ±60% starch remaining after 48 h.

SCFAs and lactic acid were quantified after fermentation (Fig. 1); no succinic acid was detected in the fermentation supernatants. Fermentation of the medium blank resulted in low levels of SCFAs (total content of ±2 μmol mg^−1^) and no lactic acid was detected (Fig. S2†). The results show that RS-3 preparations containing ≥70% RDS were rapidly fermented to ±8–9 μmol mg^−1^ acetate and ±4–4.5 μmol mg^−1^ lactate during 48 h of fermentation by this inoculum (Fig. 1A). Only minor amounts of butyrate (≤0.4 μmol mg^−1^ substrate) and no propionate were formed during fermentation of these substrates. RS-3 preparations containing 35–50% RDS were also predominantly fermented to acetate and lactate, with ≤0.7 μmol mg^−1^ butyrate and ≤0.2 μmol mg^−1^ propionate after 48 h of fermentation (Fig. 1B). After 48 h, P14-A reached a similar total amount of acids (±14.6 μmol mg^−1^) compared to samples having ≥70% RDS, likely since it was also fully degraded after 48 h of incubation (Fig. S1B†). Fermentation of N18-B and P22-B resulted in lower amounts of total acids produced (12.7 μmol mg^−1^ and 10.1 μmol mg^−1^, respectively), in line with the lower starch degradation (Fig. S1B†). The production of SCFAs and lactate during fermentation of RS-3 preparations ≥35% RDS resulted in a drop in pH to 4–4.5 after 24 h. Previously, it has also been shown that starches that are known to contain a large digestible fraction were fermented primarily to acetate and lactate, with low levels of butyrate, which consequently caused a drop in pH.^43^

Fermentation of RS-3 preparations containing ≤15% RDS (P40-B and intrinsic RS-3 (N18-A and N76-B)) resulted in different amounts of SCFAs and lactate produced after 24 and 48 h of incubation (Fig. 1C). Intrinsic RS-3 substrates (N18-A and N76-B) were predominantly fermented to acetate and butyrate (±2.0 and ±0.8 μmol mg^−1^, respectively) after 24 h of incubation, whereas fermentation of P40-B generated more acetate (±3.8 μmol mg^−1^) and also some lactate (0.6 μmol mg^−1^). The amount of additional acid produced after 48 h of fermentation was low for P40-B (±1.2 μmol mg^−1^), in line with the decreased starch degradation rate (Fig. S1C†). In contrast, the amount of additional acid produced was much higher for N18-A and N76-B (4.2 and 3.7 μmol mg^−1^, respectively), in line with the increased starch degradation rate between 24 and 48 h of incubation (Fig. S1C†). The production of SCFAs and lactate during fermentation resulted in a lower pH for P40-B (pH ± 4.7), compared to intrinsic RS-3 N18-A and N76-B (pH ± 5.3) at 24 h.

### 3.3. Partially digestible starch stimulates different microbial populations compared to intrinsic RS-3

**Fig. 3:**
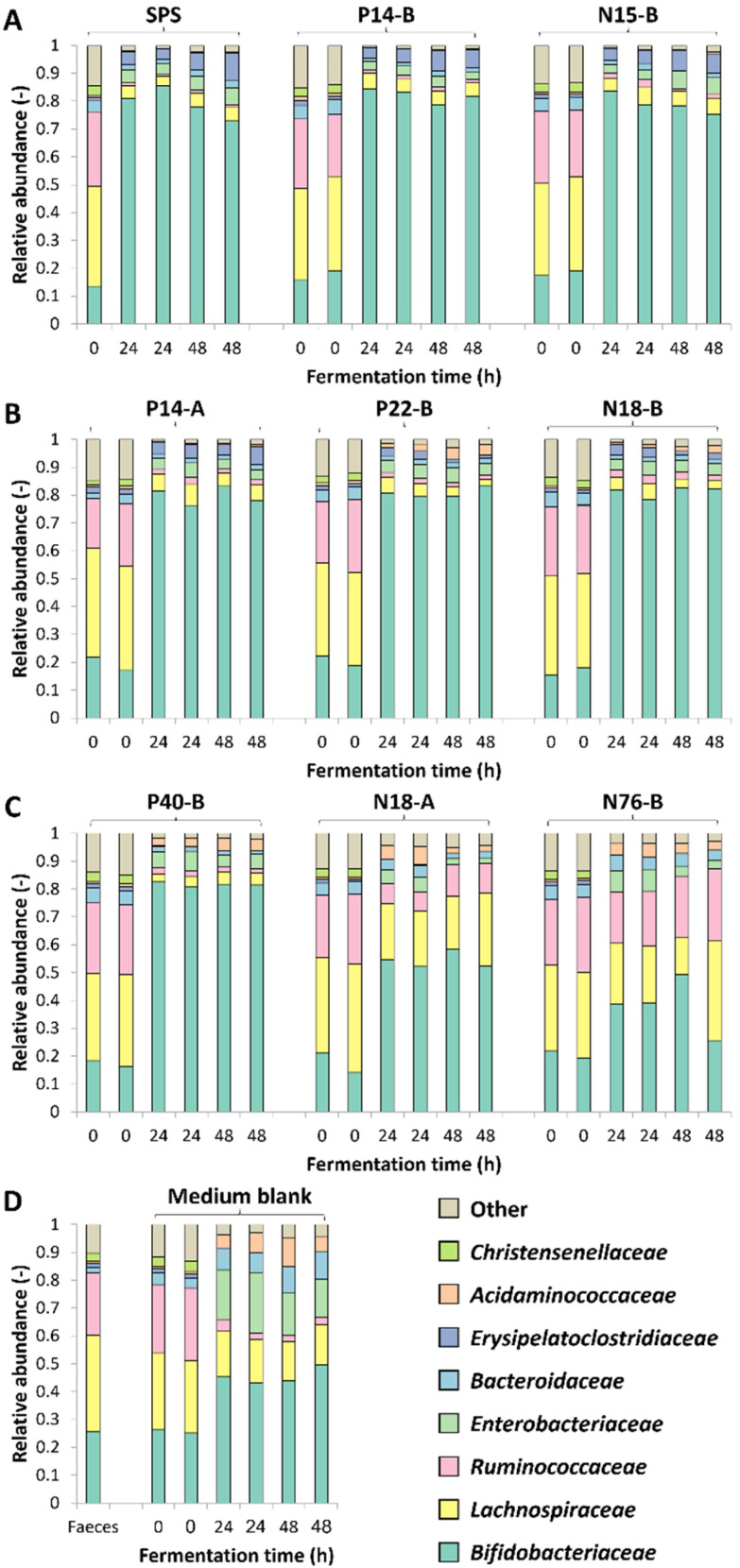
Microbiota composition in relative abundance at family level during 48 h of duplicate fermentations of RS-3 preparations by pooled adult faecal inoculum. Figure A, B, C and D represent ≥70% RDS, 35–50% RDS, ≤15% RDS and medium blank, respectively. Results of both biological duplicates are indicated.

The changes in microbiota composition during fermentation of RS-3 preparations differing in RDS content were determined using 16S rRNA gene sequencing and visualised using β-diversity analysis after centered-log-ratio (CLR) transformation of relative abundances of ASVs (Fig. 2). The PCA plot shows that t0 samples clearly differed from the samples taken after 24 and 48 h of incubation (Fig. 2A), indicating that *in vitro* fermentation selectively stimulated microbial populations compared to the initial inoculum. After 24 and 48 h of fermentation, RS-3 preparations with ≥70% RDS and 35–50% RDS fully overlapped and clustered together (Fig. 2B and C), in agreement with the similar SCFA profiles observed. Intrinsic RS-3 substrates N18-A and N76-B clearly separated from RS-3 preparations having a high fraction of digestible starch, in line with the different SCFA profiles. The microbiota in fermented P40-B, with ≤15% RDS and ±25% total digestible starch, was closer to RS-3 preparations ≥35% RDS and separated clearly from intrinsic RS-3 (N18-A and N76-B). In terms of starch degradation and SCFA production, P40-B also differed from N18-A and N76-B (Fig. S1C,†Fig. 1C), but also did not resemble fermentation of RS-3 preparations ≥35% RDS. PERMANOVA analyses confirmed a significant effect of % RDS and % RS on microbiota composition after 48 h of fermentation using Aitchison (% RDS: *P* = 2 × 10^−4^, % RS: *P* = 0.0018), unweighted UniFrac (% RDS: *P* = 1 × 10^−4^, % RS: *P* = 0.0059) and generalised UniFrac (% RDS: *P* = 7 × 10^−4^, % RS: *P* = 2 × 10^−4^) distances (Table S4†), indicating that both these factors contributed significantly to the overall microbiota composition, the presence of certain taxa and the abundance of those taxa. The microbiota compositions in relative abundance during fermentation of RS-3 preparations differing in % RDS were determined at family level and indicated that the microbiota composition at t0 obviously was quite similar for all fermentations, with around 15% *Bifidobacteriaceae*, 40% *Lachnospiraceae*, 18% *Ruminococcaceae* and 5% *Bacteroidaceae* (Fig. 3). Due to the minor relevance of the genera NK4A214 group, UCG-002, UCG-003 and UCG-005, only present in the initial inoculum and t0 samples, and due to some inconsistency in listing these genera as either *Ruminococcaceae* or *Oscillospiraceae* in literature, they were listed within *Ruminococcaceae*, since *Ruminococcaceae* and *Oscillospiraceae* are heterotypic synonyms.

After 24–48 h of fermentation of RS-3 preparations containing ≥35% RDS and P40-B, the relative abundance of *Bifidobacteriaceae* increased to ±80% (Fig. 3A–C). Fermentation of intrinsic RS-3 N18-A and N76-B (≤15% RDS) resulted in more diverse communities with an increase in relative abundance of *Bifidobacteriaceae* to 40–50%, ±20% *Lachnospiraceae* and 5% (N18-A) or 20% (N76-B) *Ruminococcaceae* (Fig. 3C). Fermentation of intrinsic RS-3 substrates N18-A and N76-B (≤15% RDS) compared to P40-B (≤15% RDS) and RS-3 preparations ≥35% RDS resulted thus clearly in different microbial populations, in line with the results shown in the PCA plots (Fig. 2). Fermentation of the medium blank, containing a minor amount of mSIEM carbohydrates and no additional carbohydrate source, resulted in an increase in relative abundance of *Bifidobacteriaceae* to 50%, and showed presence of *Lachnospiraceae*, *Enterobacteriaceae*, *Bacteroidaceae* and *Acidaminococcaceae*.

**Fig. 4:**
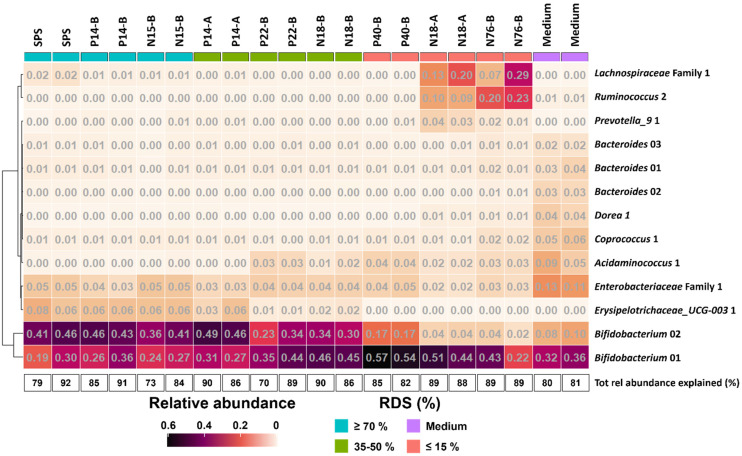
Heatmap showing the relative abundance of taxa at ASV level ≥2% in at least one individual sample after 48 h of fermentation of RS-3 preparations differing in % RDS and medium blank using pooled adult faecal inoculum. The total relative abundance explained (%) is also provided. The taxa are sorted by hierarchical clustering of Euclidean distances.

To further investigate the effect of % RDS on microbiota composition after 48 h of fermentation, we looked at ASVs explaining ≥2% of the total relative abundance within an individual sample and together explaining ≥70% of the total relative abundance (Fig. 4). The results show that the increase in relative abundance of *Bifidobacteriaceae* as shown in Fig. 3 for all samples, can be explained by the presence of primarily two ASVs (Fig. 4), both showing a co-occurrence pattern across fermentations. *Bifidobacterium* 01 was present in all fermented samples, varying from 19% for SPS to 57% for P40-B. *Bifidobacterium* 02 was especially present in fermented samples containing ≥35% RDS; the higher the amount of RDS, the higher the relative abundance of *Bifidobacterium* 02 observed. In contrast, fermented intrinsic RS-3 substrates (N18-A and N76-B) did show low (≤4%) relative abundance of *Bifidobacterium* 02. Fig. 4 shows that both *Lachnospiraceae* and *Ruminococcaceae* as present in N18-A and N76-B were each primarily explained by one ASV: *Lachnospiraceae* Family 1 and *Ruminococcus* 2, respectively. Both taxa were hardly present in the fermented medium blank, indicating that these taxa were likely involved in the fermentation of intrinsic RS-3. It is noteworthy that the microbiota composition obtained after fermentation of P40-B, an RS-3 preparation with ±15% RDS and ±10% SDS (Table 1), showed no increase in relative abundance of *Lachnospiraceae* Family 1 and *Ruminococcus* 2.

### 3.4. Soluble starch and intrinsic RS-3 differ in fermentability by various microbial populations

*In vitro* fermentation of RS-3 preparations differing in % RDS by pooled adult faecal inoculum showed differences in starch degradation, SCFA formation and microbiota composition, depending on the amount of digestible starch present. To further investigate whether the fermentability of digestible starch *versus* intrinsic RS-3 is universal or sample-specific, we incubated digestible starch (SPS) and intrinsic RS-3 (N18-A and N76-B) with individual faecal inocula obtained from 2 adults, 2 weaning 6-month-old infants and from the same 2 infants at 9–10 months old and quantified the SCFAs, lactic and succinic acid produced after 48 h of fermentation (Table S5†). Here, it is clearly shown by an increase in SCFAs, lactic and succinic acid compared to the medium blank that digestible starch was fermented by all faecal inocula, whereas intrinsic RS-3 was obviously fermented by the faecal inoculum of only one adult.

## 4. Discussion

We examined the impact of the presence of a digestible starch fraction during *in vitro* fermentation of RS-3 preparations by pooled adult faecal inoculum. Our results show that the presence of such fraction within RS-3 preparations influenced the fermentation rate, production of SCFAs and microbiota composition. *In vitro* fermentation of RS-3 preparations containing ≥25% total digestible starch within RS-3 preparations resulted in a different SCFA profile and microbiota composition compared to intrinsic RS-3 (≤20% digestible starch). By analysis of the SCFAs produced after fermentation, it was shown that digestible starch was obviously fermentable by an inoculum prepared of a pool of four adult faecal samples and by 6 individual faecal inocula tested. In contrast, as determined by additional SCFA production compared to the medium blank, intrinsic RS-3 was only fermentable by the pooled adult faecal inoculum and the inoculum of 1 individual adult faecal sample, suggesting that intrinsic RS-3 requires different microbes to be effectively degraded than digestible starch. Walker *et al.* (2011) studied the response of a diet high in RS-3 on the faecal microbiota composition of overweight men and showed by quantifying remaining starch in the faeces that some subjects were unable to ferment RS-3, likely since they had low presence of *Ruminococcus*.^59^ In a follow-up study, the ability to degrade RS was restored when faecal inoculum of one of the subjects was supplemented with *R. bromii*,^35^ clearly demonstrating the need for specialised microbes to achieve degradation of RS.

Our results indicate that even a small proportion of digestible starch, such as present within P40-B, an RS-3 preparation obtained from debranched waxy potato starch crystallised in a B-type polymorph, could steer *in vitro* fermentation. The presence of the digestible fraction within P40-B caused an initial fast fermentation and increased relative abundance of *Bifidobacterium*, after which fermentation of the resistant fraction was not possible anymore. However, in our previous study, we showed that pre-digested P40-B and pre-digested P22-B (*i.e.* with an enriched RS fraction) behaved quite similarly to intrinsic RS-3 in terms of fermentation rate, SCFA production and microbiota composition.^14^ Previously, something similar was shown for the fermentability of isomalto/malto-polysaccharides (IMMPs),^60^ a soluble starch-based dietary fibre, that consist of a long chain of linearly linked α-1,6 glucose residues, connected to a short α-1,4 linked glucose chain.^61,62^*In vitro* fermentation of these IMMPs, with and without the presence of the α-1,4 linked fraction showed that the microbiota favoured to ferment the α-1,4 linked fraction (*i.e.* the digestible fraction) over the α-1,6 linked fraction, while once the α-1,4 linked fraction was depleted, the microbiota could not ferment the α-1,6 linked fraction anymore.^60^ In contrast, when the α-1,4 linked fraction was removed prior to fermentation, the microbiota was able to fully ferment the α-1,6 linked fraction within 24 h.^60^ Also these findings indicate that presence of a small proportion of digestible starch could steer fermentation *in vitro* and thus overrule the true fermentability of the dietary fibre studied. Although we and others^43,60^ found differences in fermentability of starches depending on the presence of a digestible fraction, such differences were not always found. Warren *et al.* (2018) studied *in vitro* fermentability of RS-3 preparations from maize and potato starch using pig faecal inoculum and found a similar microbiota composition after fermentation with and without α-amylase pre-treatment.^63^ Nevertheless, the pre-treated RS-3 preparations still contained a digestible fraction, whereas different resistant starches (non RS-3) containing low digestible starch stimulated different microbial populations.^63^

As previously reported, a small proportion of digestible starch may arrive in the colon^19^ and be fermented quickly by several different gut microbes, thereby not affecting faecal microbiota composition.^20^ The resistant fraction will be further fermented by highly specialised gut microbes, that need to be present within the gut microbiome to achieve degradation.^59^ When studying fermentability of resistant starches *in vitro*, fast fermentation of the digestible fraction may cause a drop in pH resulting in lactate accumulation and limiting further cross-feeding,^64^ as observed during *in vitro* fermentation of RS-3 preparations having ≥35% RDS (Fig. 1). This is in agreement with Belenguer *et al.* (2007) who showed limited propionate and butyrate production at pH 5.2, while lactate was accumulating.^64^ Fermentation of RS-3 preparations having ≥35% RDS resulted in a relative abundance of ±80% *Bifidobacteriaceae* (Fig. 3 and 4), which are known to ferment their substrates to acetate and lactate^65^ and of which some species are known starch degraders.^66^ Microbes differ in their pH tolerance with *e.g. Bifidobacterium* being more acidic-pH tolerant than *Ruminococcus bromii*.^67^ Probably due to the acidification observed, specialised microbes able to degrade resistant starch could not grow anymore causing the slowing down of the fermentation rate as observed in Fig. S1.† This clearly illustrates that it is essential to be aware of the presence of a digestible fraction when studying fermentability of resistant starches *in vitro*. Preferably, the digestible fraction should be (partially) removed to study the prebiotic potential of resistant starches, especially when a non-pH controlled system is used.

In our study, we quantified the amount of solubilised starch during incubation of RS-3 preparations without inoculum and found especially an increased solubilisation for RS-3 preparations containing high amounts of digestible starch (Table S2†). Due to their solubility and *in vitro* digestibility, such substrates are not likely to reach the colon *in vivo*. Although useful, quantification of remaining starch during *in vitro* fermentation is often not mentioned in literature. Bui *et al.* (2020) quantified the total amount of remaining starch during *in vitro* fermentation,^27^ but did not separate the insoluble and potentially present soluble fractions. Some starches, such as Novelose® 330, a commercial RS-3 ingredient prepared from high amylose maize starch, have low solubility at room temperature.^68^ Low solubility of substrates might make separation in soluble and insoluble fractions unnecessary, assuming that insoluble starch degradation into soluble oligomers by gut microbes is slower than the uptake and further conversion to SCFAs. RS-3 prepared from debranched starches or low *M*_w_ α-1,4 glucans may partially solubilise during incubation,^58^ which makes quantification of soluble and insoluble fractions during *in vitro* fermentation necessary.

## 5. Conclusions

This study investigated the impact of the presence of a digestible starch fraction during *in vitro* fermentation of RS-3 preparations. The results showed that the presence of such digestible fraction, even when only present in a relatively small amount, may steer *in vitro* fermentation and overrule the true fermentability of the resistant starch fraction as would arrive in the colon *in vivo*. In order to evaluate the prebiotic potential of resistant starch, quantification of the remaining soluble and insoluble starch fractions after *in vitro* fermentation is beneficial to draw valid conclusions. Starch should be considered a generic carbohydrate for gut microbiota, fermentable by many different gut microbial populations, whereas this is not the case for intrinsic RS-3. Our study shows that awareness of the presence of a digestible starch fraction during *in vitro* fermentation of resistant starches is essential to evaluate its prebiotic potential *in vivo*. Depending on the research question, the digestible starch fraction within RS (preparations) should be removed, especially when a non-pH controlled system is used.

## Author contributions

Conceptualization: C. E. K., P. L. B. and H. A. S.; Investigation and methodology, writing – original draft: C. E. K.; Investigation – microbiota composition: M. F. E.; Funding acquisition: P. d. V.; Supervision: H. A. S. and J. H. B.; Writing – review & editing: M. F. E., D. K., P. d. V., E. G. Z., J. H. B., H. A. S.

## Conflicts of interest

There are no conflicts to declare.

## Supplementary Material

FO-015-D3FO01763J-s001
