# Supplementary material for: Presence of digestible starch impacts in vitro fermentation of resistant starch
Source: Food Funct. 2023 Dec 6;15(1):223–35. doi: 10.1039/d3fo01763j (PMC10760408; doi:10.1039/d3fo01763j)
Supplement: FO-015-D3FO01763J-s001 [file FO-015-D3FO01763J-s001.pdf]

This journal is © The Royal Society of Chemistry 2023

Supplementary information belonging to Klostermann et al. "Presence of digestible starch impacts *in vitro* fermentation of resistant starch"

Table S1. Starch recovery (%) in soluble and insoluble fractions of duplicate fermentations of different RS-3 preparations using pooled adult faecal inoculum during 48 h of incubation.

Sample name	Time 0 h		Time 24 h		Time 48 h	
	Soluble (%)	Insoluble (%)	Soluble (%)	Insoluble (%)	Soluble (%)	Insoluble (%)
**P14-A**	8.6 ± 0.0	83.6 ± 4.5	0.3 ± 0.0	13.7 ± 1.1	0.2 ± 0.0	1.4 ± 0.9
**P14-B**	29.7 ± 0.2	59.9 ± 1.1	0.8 ± 0.0	2.1 ± 0.1	0.6 ± 0.1	1.4 ± 0.1
**N15-B**	11.6 ± 5.0	76.8 ± 0.6	0.4 ± 0.1	9.8 ± 0.7	0.3 ± 0.2	4.0 ± 0.0
**P22-B**	13.1 ± 0.0	75.1 ± 3.2	0.0 ± 0.0	37.4 ± 0.8	0.3 ± 0.4	24.5 ± 1.3
**N18-A**	2.9 ± 0.2	87.7 ± 11.1	0.3 ± 0.0	72.5 ± 7.1	0.2 ± 0.0	30.9 ± 1.5
**N18-B**	10.7 ± 0.3	91.5 ± 0.5	0.0 ± 0.0	34.4 ± 2.8	0.1 ± 0.1	19.0 ± 3.4
**P40-B**	8.5 ± 1.6	92.1 ± 7.3	0.1 ± 0.0	69.0 ± 0.4	0.2 ± 0.1	61.4 ± 0.6
**N76-B**	1.2 ± 0.0	99.8 ± 4.5	0.1 ± 0.0	82.7 ± 8.4	0.4 ± 0.5	36.0 ± 0.3
**SPS**	83.3 ± 0.9	n.a.	0.6 ± 0.0	n.a.	0.3 ± 0.0	n.a.

Table S2. Starch recovery (%) in soluble and insoluble fractions of duplicate incubations of RS-3 preparations during 48 h.

Sample name	Time 0 h		Time 24 h		Time 48 h	
	Soluble (%)	Insoluble (%)	Soluble (%)	Insoluble (%)	Soluble (%)	Insoluble (%)
**P14-A**	8.3 ± 0.6	85.8 ± 3.9	28.2 ± 0.3	68.3 ± 3.0	28.0 ± 0.1	65.6 ± 2.0
**P14-B**	29.7 ± 0.1	62.8 ± 0.4	69.9 ± 1.8	27.8 ± 0.7	71.9 ± 4.3	28.0 ± 2.1
**N15-B**	15.1 ± 0.2	77.4 ± 1.5	32.4 ± 0.6	62.4 ± 2.6	31.4 ± 1.7	61.0 ± 1.5
**P22-B**	11.8 ± 0.4	86.6 ± 0.3	20.2 ± 0.5	76.2 ± 6.9	20.0 ± 0.8	79.3 ± 6.4
**N18-A**	1.9 ± 0.0	90.8 ± 5.4	6.5 ± 0.7	83.4 ± 9.1	6.7 ± 0.2	88.4 ± 2.7
**N18-B**	9.6 ± 0.3	94.0 ± 6.2	20.4 ± 1.4	81.9 ± 2.5	20.7 ± 0.8	79.1 ± 5.5
**P40-B**	10.2 ± 0.1	88.3 ± 4.2	15.3 ± 1.0	83.8 ± 5.7	15.7 ± 0.2	79.7 ± 13.7
**N76-B**	0.2 ± 0.0	94.7 ± 1.6	0.9 ± 0.3	101.3 ± 1.0	0.5 ± 0.1	98.9 ± 3.0
**SPS**	100.6 ± 12.0	n.a.	99.1 ± 0.9	n.a.	100.0 ± 1.3	n.a.

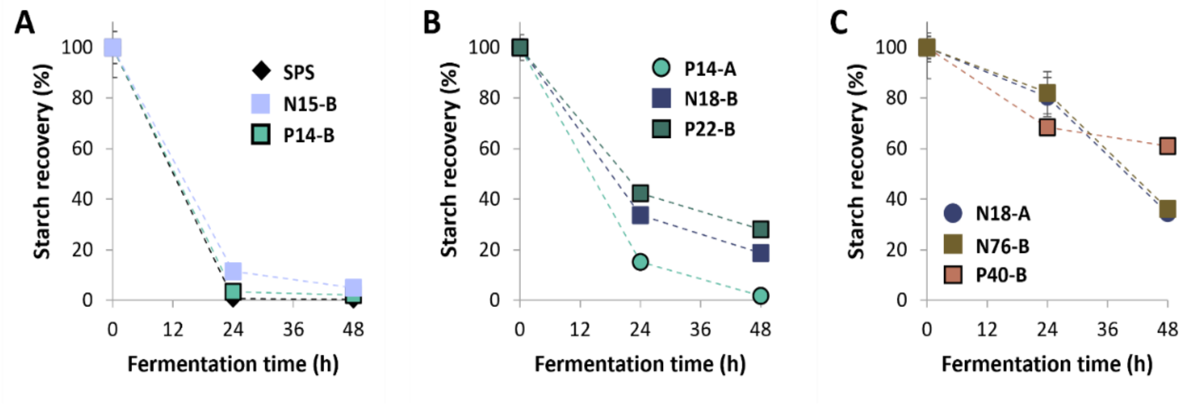

Figure S1. Starch recovery (soluble + insoluble starch) during 48 h of fermentation of RS-3 preparations by pooled adult faecal inoculum, normalised for the total starch content at t0. Figure A, B and C represent RS-3 preparations containing ≥ 70 % RDS, 35-50 % RDS or ≤ 15 % RDS, respectively. The average of biological duplicates is shown. Standard deviations might be smaller than the marker used.

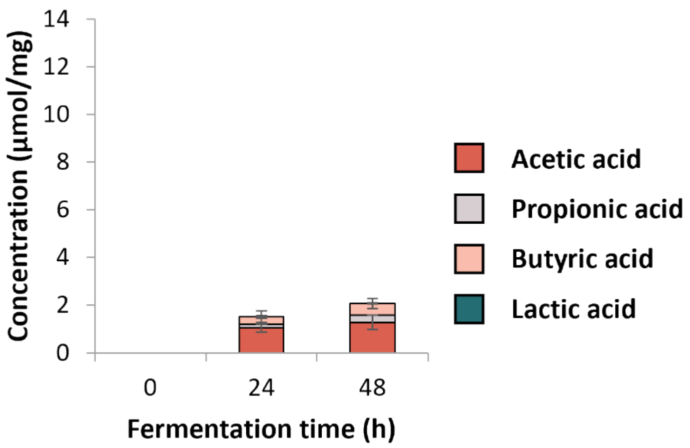

Figure S2. SCFA and lactic acid content (µmol/mg substrate) during 48 h of incubation of the medium blank with pooled adult faecal inoculum. The average of biological duplicates is shown.

Table S3. PERMANOVA of RDS, SDS and RS on Aitchison distances of ASVs, Unweighted UniFrac and Generalized UniFrac distances on microbiota compositions of fermented RS-3 preparations after 24 h of incubation.

Time 24 h	Variable	R^2	Pr
**Aitchison**	RDS	0.27226	1e-04
	SDS	0.12272	0.0288
	RS	0.1996	0.0017
**Unweighted UniFrac**	RDS	0.41345	1e-04
	SDS	0.18455	0.011
	RS	0.21107	0.0048
**Generalized. UniFrac**	RDS	0.49388	3e-04
	SDS	0.13972	0.0568
	RS	0.37032	0.001

Table S4. PERMANOVA of RDS, SDS and RS on Aitchison distances of ASVs, Unweighted UniFrac and Generalized UniFrac distances on microbiota compositions of fermented RS-3 preparations after 48 h of incubation.

Time 48 h	Variable	R^2	Pr
**Aitchison**	RDS	0.28846	2e-04
	SDS	0.10182	0.0636
	RS	0.19391	0.0018
**Unweighted UniFrac**	RDS	0.40323	1e-04
	SDS	0.23791	0.0023
	RS	0.19977	0.0059
**Generalized UniFrac**	RDS	0.39336	7e-04
	SDS	0.09752	0.1143
	RS	0.33754	2e-04

Information S1.

In vitro batch fermentations using individual faecal inocula were performed as previously described (Klostermann et al. (2023)) with minor modifications. Individual faecal inocula were prepared by diluting faecal slurries to 10 mg/mL in mSIEM. N18-A and N76-B were weighed in duplicate in sterile 5 mL serum bottles (± 10 mg dry weight) and 1.8 mL mSIEM and 0.2 mL inoculum were added to reach final substrate concentrations of ± 5 mg/mL. Also samples with SPS (at ± 5 mg/mL), substrate blanks (± 5 mg/mL, without inoculum) and medium blanks (without additional substrate) were prepared. The serum bottles were capped with butyl rubber stoppers and incubated at 37 °C, 100 rpm for 0 and 48 h.

Sampling was performed as described in section 2.2. SCFAs were analysed according to Klostermann et al. (2023). The microbiota composition of the faecal samples was obtained as described in section 2.5 (Figure S2).

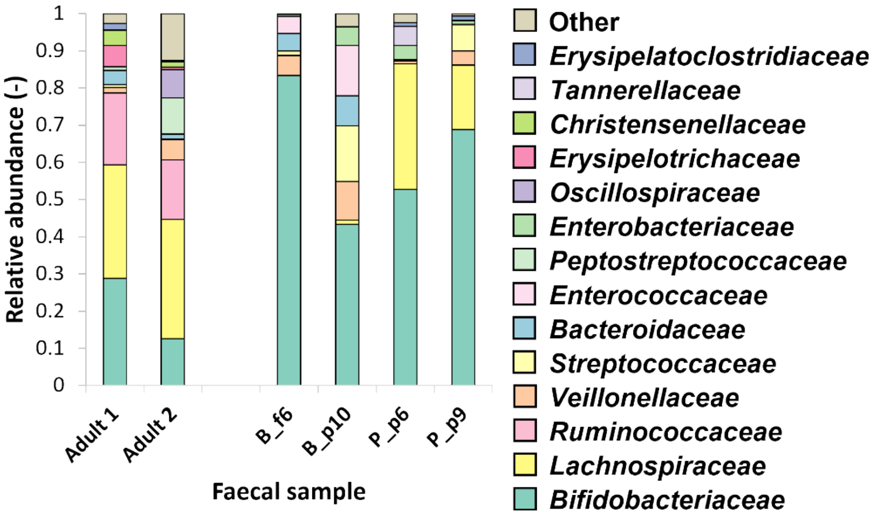

Figure S3. Microbiota composition (relative abundance) at family level of adult and infant faecal samples at 6 and 9-10 months old.

Table S5. SCFAs and other organic acids (µmol/mg substrate) produced during 48 h of incubation of intrinsic RS-3 N18-A and N76-B, SPS and medium blank with different faecal inocula. The average of biological duplicates is shown.

Inoculum	Substrate	Total SCFA (µmol/mg)	A : P : B	L (µmol/mg)	S (µmol/mg)
**A1**	N18-A	4.1 ± 0.2	0.5 : 0.1 : 0.3	n.d.	0.5
	N76-B	3.7 ± 0.4	0.5 : 0.2 : 0.3	n.d.	n.d.
	SPS	8.2 ± 0.1	0.9 : 0.0 : 0.1	2.9	0.4
	Medium blank	3.0 ± 0.1	0.5 : 0.2 : 0.3	n.d.	n.d.
**A2**	N18-A	7.1 ± 1.1	0.5 : 0.1 : 0.4	n.d.	n.d.
	N76-B	6.1 ± 0.1	0.6 : 0.1 : 0.3	0.1	n.d.
	SPS	8.1 ± 0.0	0.9 : 0.0 : 0.1	3.5	0.1
	Medium blank	3.3 ± 0.2	0.6 : 0.2 : 0.2	n.d.	n.d.
**B_f6**	N18-A	3.6 ± 0.1	0.6 : 0.4 : 0.0	n.d.	n.d.
	N76-B	1.9 ± 0.2	0.8 : 0.2 : 0.0	n.d.	0.2
	SPS	9.4 ± 0.1	0.9 : 0.1 : 0.0	0.0	n.d.
	Medium blank	2.0 ± 0.0	0.7 : 0.3 : 0.0	n.d.	n.d.
**B_p10**	N18-A	3.4 ± 0.1	0.5 : 0.5 : 0.0	n.d.	n.d.
	N76-B	2.6 ± 0.0	0.5 : 0.5 : 0.0	n.d.	n.d.
	SPS	8.7 ± 0.4	0.7 : 0.3 : 0.0	0.1	n.d.
	Medium blank	2.4 ± 0.0	0.5 : 0.5 : 0.0	n.d.	n.d.
**P_p6**	N18-A	2.7 ± 0.1	0.7 : 0.3 : 0.0	0.2	0.3
	N76-B	1.8 ± 0.1	0.7 : 0.3 : 0.0	0.1	0.2
	SPS	5.6 ± 0.2	1.0 : 0.0 : 0.0	3.9	0.4
	Medium blank	1.9 ± 0.1	0.7 : 0.3 : 0.0	0.1	0.1
**P_p9**	N18-A	4.5 ± 0.3	0.5 : 0.4 : 0.1	n.d.	n.d.
	N76-B	3.4 ± 0.0	0.5 : 0.4 : 0.2	n.d.	n.d.
	SPS	2.4 ± 0.0	1.0 : 0.0 : 0.0	7.0	n.d.
	Medium blank	3.1 ± 0.0	0.5 : 0.3 : 0.2	n.d.	n.d.

A1 and A2 refer to adult faecal inocula, whereas B_f6 & B_p10 (infant B at 6 and 10 months old) and P_p6 & P_p9 (infant P at 6 and 9 months old) refer to infant faecal inocula. The initial microbiota compositions are shown in Figure S2. Total SCFAs include acetic, propionic and butyric acid, whereas A : P : B give the ratio between these acids. L and S refer to lactic and succinic acid, respectively. n.d. = non-detected.
